# Editorial: Advances in approaches for function-preserving gastric cancer surgery

**DOI:** 10.3389/fsurg.2023.1335914

**Published:** 2024-01-26

**Authors:** Ruiguang Ma, Zhibo Yan, Nadia M. Hamdy, Xianquan Zhan, Zhen Li

**Affiliations:** ^1^ Department of Gastroenterology, Qilu Hospital of Shandong University, Jinan, China; ^2^ Department of Gastrointestinal Surgery, Qilu Hospital of Shandong University, Jinan, China; ^3^ Department of Biochemistry, Faculty of Pharmacy, Ain Shams University, Cairo, Egypt; ^4^ Medical Science and Technology Innovation Center, Shandong First Medical University, Jinan, China

**Keywords:** function-preserving gastric cancer surgery, advances and challenges, postoperative quality of life, gastric cancer, laparoscopic gastrectomy

**Editorial on the Research Topic** Advances in approaches for function-preserving gastric cancer surgery

Gastric cancer is a common malignancy and a leading cause of cancer death globally. In the past, patients with early gastric cancer (EGC) are generally treated with standard gastrectomy. Although the survival rate in patients with EGC is more than 95%, it may cause loss of gastric functions and lower postoperative quality of life (QoL). Due to the low incidence of lymph node metastasis and the favorable prognosis in EGC, function-preserving gastrectomy, with an adequate range of gastric resection and minimal lymphadenectomy, could improve the patient's QoL. Surgical resection is a conventional treatment for EGC. Pylorus-preserving gastrectomy and proximal gastrectomy (PG) represent the two main function-preserving surgical procedures for GC. Recently, minimally invasive approaches, such as endoscopic therapy or laparoscopic gastrectomy (including robot-assisted surgery), have been widely applied for EGC treatment. This Research Topic aims to provide a collection of reports to present novel techniques and studies on the oncological safety and effectiveness of the approaches for function-preserving gastric cancer surgery.

PG is an alternative to total gastrectomy for cT1N0 EGC in the upper third of the stomach. In PG, the rate of complications, such as reflux esophagitis and anastomotic stenosis, was markedly higher compared with total gastrectomy. As a result, many scholars have explored and improved the digestive tract reconstruction methods during PG to reduce the incidence of reflux esophagitis and improve the postoperative QoL of patients. There needs to be more studies on the QoL of patients after proximal gastrectomy. Meng et al. demonstrated that tube esophagogastric anastomosis is a safe and feasible method of gastrointestinal reconstruction with more advantages over traditional esophagogastric anastomosis in restoring postoperative gastrointestinal function and reducing reflux. Of note, after the propensity score matched, they proved that the application of indocyanine green tracer technique in laparoscopic radical proximal gastrectomy has positive significance. Indocyanine green fluorescence imaging helps visualize lymphatic flow during sentinel lymph node mapping, even in laparoscopic surgery.

Similarly, Xu et al. conducted a multicenter, retrospective cohort study in patients with adenocarcinoma of the esophagogastric junction (AEG). They compared laparoscopic proximal gastrectomy with double-tract reconstruction (LPG-DTR) and laparoscopic proximal gastrectomy with tube-like stomach reconstruction (LPG-TLR) in terms of perioperative recovery, postoperative anti-reflux effect, nutrition-related index changes, and QoL. Patients in both groups had a similar postoperative QoL. The incidence of reflux esophagitis did not reach a statistical difference in LPG-TLR patients and LPG-DTR patients. However, LPG-DTR provides better nutrition status than LPG-TLR in AEG patients.

The management of esophagogastric anastomotic stenosis is challenging, which is a relatively common complication after PG. Current endoscopic management is the standard therapeutic approach, encompassing bougie or balloon dilation, stent placement, and needle-knife stricturotomy, with or without intralesional steroid injection, to reduce the inflammatory response and prevent restenosis. Tian et al. reported an AEG patient who successfully underwent endoscopic stricturotomy after LPG with valvuloplasty. They stated that utilizing endoscopic stricturotomy to treat anastomotic stenosis after PG with valvulopasty can be considered a safe option and should be performed in well-established centers of expertise.

As GC progresses, patients with tumors located in the lower part of the stomach may develop pyloric outlet obstruction, which worsens the health conditions and makes treatment even more challenging. Wang et al. compared complication rates and the length of hospital stay between patients with and without pyloric outlet obstruction in open and laparoscopic groups. Laparoscopic surgery provided a lower overall complication rate, shorter postoperative length of hospital stay, and more harvested lymph nodes over open surgery.

Currently, surgery combined with postoperative chemotherapy for GC patients improves the prognosis in early-stage GC; however, the prognosis of intermediate and advanced gastric cancer remains unsatisfactory. In recent years, immune checkpoint inhibitors have shown promising prospects for treating advanced GC. Yuan et al. summarized the efficacy and safety data of medication in the latest prospective studies in systematic review and meta-analysis. They found that PD-1/PD-L1 inhibitors combined with neoadjuvant chemotherapy for locally advanced, resectable gastric or gastroesophageal junction adenocarcinoma were well tolerated and may confer therapeutic advantages.

Collectively, the collection of research in this Research Topic provides advances in approaches for function-preserving gastric cancer surgery. There are several comparative studies in this column. In the future, the comparative data may allow clinical physicians to practice personalized and value-based care. When patients consider their best approach, they can make informed decisions about their choice of procedure based on the effectiveness and oncological safety. We are aware we did not manage to cover all aspects of the issue of function-preserving gastric cancer treatment. Also, extensive multicenter studies with sufficiently long follow-ups of large numbers of patients are needed and will help to validate these critical clinical questions. However, we believe this Research Topic has shed light on many elements and will be helpful for further research on the issue of function-preserving gastric cancer treatment in GC patients.

